# Insecticidal and Genotoxic effects of some indigenous plant extracts in *Culex quinquefasciatus* Say Mosquitoes

**DOI:** 10.1038/s41598-020-63815-w

**Published:** 2020-04-22

**Authors:** Muhammad Zulhussnain, Muhammad Kashif Zahoor, Hina Rizvi, Muhammad Asif Zahoor, Azhar Rasul, Aftab Ahmad, Humara Naz Majeed, Amer Rasul, Kanwal Ranian, Farhat Jabeen

**Affiliations:** 1 Department of Zoology, Government College University Faisalabad, Faisalabad, Pakistan; 2 Department of Environmental Sciences & Engineering, Government College University Faisalabad, Faisalabad, Pakistan; 3 Department of Microbiology, Government College University Faisalabad, Faisalabad, Pakistan; 4 Department of Biochemistry/US-Pakistan Center for Advance Studies in Agriculture and Food Security (USPCAS-AFS), University of Agriculture Faisalabad, Faisalabad, Pakistan; 5 Department of Biochemistry, Government College Women University, Faisalabad, Pakistan; 6 Department of Entomology, University of Agriculture Faisalabad, Faisalabad, Pakistan

**Keywords:** Agricultural genetics, Entomology

## Abstract

Five different weed plants viz. *Convulvulus arvensis, Chenopodium murale, Tribulus terrestris, Trianthema portulacastrum*, and *Achyranthes aspera* were investigated for their entomocidal and genotoxic effects against *Culex quinquefasciatus* mosquitoes. High mortality was observed at 72 hours in a dose dependent manner. Among all the tested plants, *A. aspera* was found highly significant which showed 100% mortality at 250 ppm after 72 hours with LC_50_ of 87.46, 39.08 and 9.22 ppm at 24, 48, respectively. In combination with *Bacillus thuringiensis israelensis* (*Bti*); *A. aspera* also caused 100% mortality at 250 ppm concentration after 72 hours (LC_50_ 8.29 ppm). Phytochemical analysis of all the tested weed plants showed the presence of flavonoids, saponins, tannins, steroids, cardiac glycosides, alkaloids, anthrequinones and terpenoids. Random Amplification of Polymorphic DNA-Polymerase chain reaction (RAPD-PCR) and comet assay were performed to assess the genotoxic effect of *A. aspera* but no change in DNA profile was observed. Furthermore, FTIR showed the presence of phenolic compounds in *A. aspera* extract. It is suggested that certain phenolic compounds such as flavonoids modulate the enzymatic activity and, hence, cause the death of larvae of *Cx. quinquefasciatus*. Altogether, current study would serve as an initial step towards replacement of synthetic insecticides to plant-microbe based biopesticide against *Culex* mosquitoes in future.

## Introduction

Mosquitoes are reported to cause nuisance to humans and transmit several viral and protozoan diseases of public health concern worldwide. These are female mosquitoes which make a bite during their search for blood meal before oviposition which thus, increases their tendency to transmit several diseases including malaria, filariasis, dengue fever, japanese encephalitis, chikungunya, zika virus and yellow fever. These diseases make life at risk of millions of people particularly in subtropical/tropical world^1,2^. Of various mosquito species, *Cx. quinquefasciatus* transmits various diseases i.e., West Nile virus, Japanese encephalitis, filariasis, bancroftian filariasis (*Wuchereria bancrofti*), St. Louis encephalitis, and avian malaria^3^. In southern United States, St. Louis virus and West Nile virus (WNV) were transmitted by *Cx. quinquefasciatus*^4,5^. Almost 120 million people are affected annually only by lymphatic filariasis, whereas 1.3 billion are at risk resulting in nearly $1.3 billion loss of productivity per year^6^. Similarly, three billion individuals are at risk of being infected by Japanese encephalitis with 30,000–50,000 reported cases every year in disease endemic areas^7^. Besides disease transmission in humans; *Cx. quinquefasciatus* is also responsible for transmitting several diseases to livestock and companion animals viz. Rift Valley fever, canine dirofilariasis (dog heartworm), avian malaria, avian pox, and West Nile encephalitis which lead to high mortalities or decreased productivity^8^.

To avoid proliferation of mosquito borne diseases; mosquito control is necessary which is essentially performed through using chemical insecticides. Many synthetic agents such as organochlorine and organophosphate compounds have been developed and employed with a considerable success despite of therein including toxicity to non-targeted organisms and fostered sometimes severe environmental and human health concerns^9^. The continuous use of synthetic insecticide results in the development of resistance in mosquitoes. Furthermore non-degradable nature of synthetic insecticide causes biomagnification which makes the situation overall more worst^10–12^. Collectively, the current status situation urges to find out environment friendly, cost-effective, biodegradable and target specific insecticides against mosquitoes. Although, an eco-friendly alternative approach such as biological control became the central focus to exploiting certain natural enemies including predatory and parasitic species; however, mosquito control always remained a very serious issue.

Plant extracts have been reported for the control of mosquitoes^13,14^ and recently, weed plant extracts are being investigated^15^. Nevertheless, the plant extracts are biodegradable, non-hazardous and have been found active against a number of insect pests^16^. Previously, it was also focused on the commercial use of plant extracts as potent insect-control agents^17,18^. Subsequently, plants derived secondary metabolites are responsible for defense to survive against selection pressure of herbivore predators and different environmental factors. Numerous phytochemical groups like alkaloids, terpenoids, steroids, phenolics and essential oils extracted from various plants are reported as potent insecticides^16,19^. For instance, *Salvia ballotiflora* contains 37 different compounds with β-caryophyllene and caryophyllene oxide as main components and resulted in 80% larval mortality of *Cx. quinquefasciatus*^20^. The phytochemical analysis showed the presence of aromadendrene, naphthalene, α-humulene, caryophyllene oxide, caryophyllene, phenol, 4-(3,7-dimethyl-3-ethenylocta-1,6- dienyl) and methyl hexadecanoate compounds in *Psoralea corylifolia* and caused DNA damage in *Cx. quinquefasciatus*^21^. Though, first report of genomic alterations using RAPD-PCR fingerprinting was reported by Lalrotluanga & Gurusubramanian^22^ in mosquito larvae treated with various plant extracts. Variations in DNA band were observed when *P. ferulacea* essential oil-treated larvae of *E. kuehniella* and were subjected to DNA damage analysis by RAPD assay^23^. A significant DNA damage has been reported in *Aedes aegypti* due to *A. aspera*^24^. Similarly, DNA damage was also shown in *Cx. quinquefasciatus* larvae treated with *Curcuma longa* and *Melia azedarach* plant extracts^21^.

It has also been described that the enzymatic profiles are modulated in response to natural oils from plants^25^. Esterases, a major detoxifying enzyme in insects, are involved in detoxification of insecticides^26^. Plant extracts are described as AChE inhibitors^10,27^. Subsequent changes in Phosphatases enzyme activity are also reported in insects^25,26^. Additionally, the discovery of highly toxic bacterial strains against dipteran larvae such as *Bacillus thuringiensis israelensis* (*Bti*) and *Bacillus sphaericus* Neide (*Bs*) provided an option to incorporate in mosquito control programs as a potent biolarvicides around the world^28–30^.

Weeds are undesired flora which compete with crop plants for food space and nutrients and provide the alternative food for pests. In attempting to eradicate these weeds; why not this is appropriate to use them for insect control and to develop an environmentally safe insect-control agent in future^15^. Therefore, the current study was designed to evaluate the entomocidal impact of different weed plant extracts individually and in combination with microbial strains along with the genotoxic effect of weed plant extracts against *Cx. quinquefasciatus* mosquitoes.

## Materials and methods

### Collection and Rearing of *Culex quinquefasciatus*

The adults and larvae of *Cx. quinquefasciatus* were collected from main drain of Government College University, Faisalabad. The mosquitoes were then reared in plastic and enamel tray with tap water under standard conditions (26 ± 1 °C, 60 ± 10% RH with 12 hours day/night cycle) in Lab, Department of Zoology in Government College University Faisalabad. The newly emerged larvae were fed on grounded Fish Food and 5–8 days old larvae were fed on two tablets of Purina Cat Food daily. The grounded Cat Food was added after 8 days. Tray was kept in insect cage after the formation of pupae. After 2 days, adults were emerged from the pupae. A beaker having cotton soaked in 10% sugar solution was kept to provide sugar contents to adult mosquitoes. For blood feeding to female *Cx. quinquefasciatus* mosquitoes, an albino rat in a small cage was left overnight in the rearing cage^31^.

### Collection of microbes

The *Psuedomonas aeruginosa* isolate was procured from Department of Microbiology whereas *Bacillus thuringiensis israelensis* (*Bti*) was purchased from Summit^®^, USA. *P. aeruginosa* was isolated from burn wound samples using *Psuedomonas* agar (oxoid, UK). The bacterial count was adjusted to 1 × 10^8^ colony forming unit/ml (CFU/ml). This stock solution was used to prepare different ppm solutions. Similarly, 100% solution was prepared by dissolving 10% dunk of *Bti* in 10 ml distilled water. Then 1000 ppm stock solution was prepared by dissolving 10 µl of this *Bti* solution in 90 µl distilled water. This stock solution was used for further dilutions.

### Collection and identification of weed plants

Weed plants were collected from different areas of District Faisalabad and identified from Department of Botany, Government College University Faisalabad and Department of Botany, University of Agriculture Faisalabad, Pakistan. The used weed plants for extraction are listed in Table 1.

**Table 1:** List of weed plants used for oil extraction.

Sr #	Common name	Scientific Name	Collection Area	GPS Coordinates
1	Lilly	*Convulvulus arvensis*	Sammundri	31.0691°N, 72.9361°E
2	Krund	*Chenopodium murale*	Sammundri	31.0691°N, 72.9361°E
3	Bhakhra	*Tribulus terrestris*	Satyana	31.2047°N, 73.1711°E
4	Itsit	*Trianthema portulacastrum*	Satyana	31.2047°N, 73.1711°E
5	Puthkanda	*Achyrnathes aspera*	Tandlianwala	31.0368°N, 73.1379°E

#### Petroleum ether extraction of weed plants

Weed plants were cleaned by washing with clean water and shade-dried in Lab for a week. Dried plants were crushed in small pieces for further grinding. Crushed dried plants were again oven dried at 60 °C for 20 minutes and grinded in electrical grinder to obtain powder form. Extracts were obtained from 15 g of powder in 150 ml of petroleum ether of each weed plant using Soxhlet apparatus after several rotations in 8 hours. The extracts were then stored in clean and air tight bottles at 4 °C^15^.

### Mortality bioassay test

Different concentrations of each weed plant extract from 10 ppm to 250 ppm were used to perform the bioassay test following WHO protocol^32^. Twenty larvae of *Cx. quinquefasciatus* were treated with different concentrations of each extract individually and in combination with microbes (1:1 volume); *P. aeruginosa* and *Bacillus thuringiensis israelensis* (*Bti*) along with control group and group treated with different concentrations of permethrin (10, 20, 30, 50, 100, 150 and 250 ppm) in water. Three replicates were performed for each test and the mortality data was recorded at 24, 48 and 72 hours of post treatment^16^. Immovable larvae were considered as dead and removed to prevent decomposition which might cause the mortality of other intact alive larvae. The dead larvae were stored in ethanol in 1.5 ml eppendorf tubes to further determine the DNA damage by RAPD PCR and comet assay.

### Esterases and phosphatases enzyme assay

The larvae of *Cx. quinquefasciatus* mosquitoes were thoroughly washed with distilled water and adhering water was removed using blotting paper. The larvae were homogenized using ice-cold Sodium Phosphate buffer (20 mM. pH 7.0) with the help of Teflon hand homogenizer. The homogenate was centrifuged at 8000 × g and 4 °C for 20 minutes using centrifuge machine, SIGMA, Germany. The supernatant was used for the estimation of Esterases and Phosphatases (AChE = acetylcholinesterase, AcP = acid phosphatases, AkP = alkaline phosphatases, α-Carboxyl = α-Carboxylesterases and β-Carboxyl = β-Carboxylesterases). All the solutions and glassware used for homogenization were kept at 4 °C prior to use and the homogenates were held on ice until used for assays. The protocols for enzymatic assays as already described Younes *et al*.^33^ and Sultana *et al*.^15^ were followed.

### Phytochemical analysis of weeds extracts

Phytochemical analysis of five tested weed plant extracts were performed in order to detect the chemical constituents as described by Harborne^34^, Trease and Evans^35^ and Sofowara^36^.

### Fourier transform infrared spectroscopy (FTIR) analysis

The functional groups of active components in the extracts of weed plants were identified using FTIR spectrometer (Bruker Tensor II) on the basis of vibrational frequencies between atomic bonds. The extracts were chilled at −80 °C followed by lyophilization to obtain the IR spectrum of lyophilized extract (Alpha, Bruker, California, USA). FTIR spectra were measured in the frequency ranges from 400–4000 cm^−1^ sample by scanning the sample. The samples were run in triplet form^37^.

### DNA extraction

The stored samples of mosquito larvae were homogenized in 300 µl lysis buffer (0.4 M NaCl, 2 mM EDTA, and 10 mM Tris-HCL pH 8.0), 100 µl Proteinase K (100 mg/µl) of BIOSHOP, Canada and 20% sodium dodecyl sulphate (SDS). The homogenate was incubated at 55 °C for one hour, then, 300 µl of 5 M NaCl was added and vortexed for few seconds. The mixture was centrifuged at 13,000 rpm for 10 minutes. The DNA from supernatant was precipitated by adding ice cold ethanol in equal volume, and kept at −20 °C for 1 hour and afterwards recovered by centrifugation. The DNA pellet was air dried and resuspended in D_3_H_2_O^38–40^.

The optical absorbance of each sample was calculated by measuring the absorption at 260 nm wavelength of UV light using spectrophotometer of HITACHI, Japan. DNA Concentration was calculated as:

$${\rm{DNA}}\,{\rm{Conc}}.\,\mu {\rm{g}}/\mu {\rm{l}}={\rm{Dilution}}\,{\rm{fold}}\times {\rm{absorbance}}\,{\rm{at}}\,260\,{\rm{nm}}$$

### RAPD-PCR amplification

A total of five RAPD primers (GenLink: A-03, A-04, A-06, A-18 and C-04; Supplementary Table) were selected to amplify the mosquitoes genomic DNA following the PCR conditions as described by Bibi *et al*.^39^ and Zahoor *et al*.^41^.

### Haemocyte collection

*Cx. quinquefasciatus* haemocytes were collected according to Irving *et al*.^42^. The collected larvae from trail beaker were first washed with distilled water, sterilized in 5% bleach and dried. The cuticle was removed with two fine forceps. The haemolymph and haemocytes were taken in microcentrifuge tubes. The pooled haemolymph was centrifuged at 300 × g and 4 °C for 10 minutes, the supernatant was discarded and the pellet was resuspended in 20 µL of cold PBS.

### Comet assay

The comet assay was performed according to Singh *et al*.^43^ with minor modifications. The cell samples were carefully suspended in 140 µL of 0.75% LMA (Low melting agar) and then layered onto microscope slides coated with 150 µL of 1% NMA (Normal melting agar) and dried at room temperature. The two gels on each slide were mounted, covered with a coverslip and put at 4 °C for10 minutes to let solidify the gel. The coverslip was immediately removed after agarose solidification. The slides were immersed in a cold fresh lysis solution (2.5 M NaCl, 100 mM EDTA pH 10, 10 mM Tris, 1% Triton X-100 and 5% DMSO) for 2 hours at 4 °C in a dark chamber. The slides were placed in a horizontal gel electrophoresis tank filled with cold electrophoretic buffer (1 mM Na_2_EDTA and 300 mM NaOH, pH 13) for 25 minutes for DNA unwinding. The electrophoresis was performed in the same buffer for 20 min at 25 V and 300 mA (0.73 V/cm). After electrophoresis, the slide was washed twice with 0.4 mM Tris (pH 7.5) for 5 minutes to neutralize the slides. The slides were stained with 20 µL of DAPI (1 µg/mL) per gel and examined at 400× magnification with Komet 5.5 Image Analysis System fitted to an Olympus BX50 fluorescence microscope equipped with 590 nm barrier filter and 480–550 nm wide band excitation filter. One hundred randomly selected cells (50 cells per two replicate slides) per treatment were analyzed^21,44,45^.

#### Data analysis

Probit Analysis program (version 1.5) was used to determine the LC_50_ between concentration and percent mortality at various concentrations of plant extract and bacteria^46^. Abbot’s formula was used to analyze the data of mortality obtained through bioassay tests. The corrected mortality data was subjected to ANOVA using Statistica 13.0 for Windows^15^. The means were separated using Tuckey’s HSD (Honest Significant Difference) test at a significance level of 0.05. A value of p < 0.05 was considered statistically significant^14,47,48^. PCR products were analyzed using gel electrophoresis and the genetic data were analyzed using POPGENE software^40^. For comet assay, the DNA damage in cells of *Cx. quinquefasciatus* larvae was assessed by two distinct types of DNA damage measurements: the length of DNA comet tail and the percentage of fragmented DNA present in the tail after electrophoresis^21^.

## Results

### Mortality assay of *culex quinquefasciatus* using weed plant extracts at various concentrations and different time intervals

The comparison of insecticidal activity of five different weed plant extracts against larvae of *Cx. quinquefasciatus* using different concentrations at various exposure intervals is shown in Table 2. High mortality was obtained by all the weed plant extracts after 72 hours exposure time compared to *Azadirachta indica* (Neem) extract at 250 ppm concentration. But as compare to synthetic pesticide (Permethrin) only *T. terrestris* and *C. murale* showed the low mortality among all weed plants extracts. The highest mean mortality was shown by *Achyrathes aspera* (42.76, 61.28, 66.93, 78.11, 88.22, 93.43 and 100%) followed by *C. arvensis* (34.34, 42.76, 44.67, 49.50, 62.96, 71.85 and 88.22%) and *T. portulacastrum* (32.66, 44.44, 49.86, 62.96, 74.75, 79.06% and 88.22%) at 10, 20, 30, 50, 100, 150 and 250 ppm concentrations, respectively. *T. terrestris* showed low mean mortality (15.82, 29.29, 31.78, 34.34, 52.86, 59.32 and 73.06%) whereas, lowest mortality among all the five extracts was observed with *C. murale* (15.82, 20.88, 22.68, 27.61, 42.76, 49.82 and 64.65%) at 10, 20, 30, 50, 100, 150 and 250 ppm concentrations, respectively. Permethrin *s*howed (13.51, 18.01, 39.28, 43.36, 51.95, 67.14 and 76.46%) mortality at the same concentrations, respectively. *A. indica* (Neem) extract showed lowest mortality when compared to all the tested plants and Permethrin (Table 2). The overall results indicated that mortality was increased with increased extract concentrations.

**Table 2:** Mean mortality of *Culex quinquefasciatus* by different weed extracts at various concentration and different time intervals.

Treatment	Conc.	F Value	df	P value	Mean mortality with different time intervals		
					24 hours	48 hours	72 hours
Z1	10 ppm	41.91	2	<0.05	6.67 ± 1.67c	16.67 ± 1.67b	34.34 ± 2.92a
	20 ppm	88.23	2	<0.05	11.67 ± 1.67c	23.33 ± 1.67b	42.76 ± 1.68a
	30 ppm	68.49	2	<0.05	15.68 ± 1.68c	28.34 ± 1.68b	44.67 ± 1.67a
	50 ppm	59.05	2	<0.05	20.00 ± 2.89c	36.67 ± 1.67b	49.50 ± 0.00a
	100 ppm	78.87	2	<0.05	25.00 ± 2.89c	48.33 ± 1.67b	62.96 ± 1.68a
	150 ppm	92.58	2	<0.05	29.88 ± 2.88c	55.67 ± 1.67b	71.85 ± 1.68a
	250 ppm	134.03	2	<0.05	38.33 ± 1.67c	65.00 ± 2.89b	88.22 ± 1.68a
	10 ppm	14.13	2	<0.05	3.33 ± 1.67b	8.33 ± 1.67b	15.82 ± 1.68a
	20 ppm	8.61	2	<0.05	11.67 ± 1.67b	13.33 ± 1.67b	20.88 ± 1.68a
	30 ppm	7.79	2	<0.05	12.38 ± 1.67b	15.67 ± 1.67b	22.68 ± 1.68a
Z2	50 ppm	27.27	2	<0.05	13.33 ± 1.67c	20.00 ± 0.00b	27.61 ± 1.68a
	100 ppm	61.98	2	<0.05	16.67 ± 1.67c	26.67 ± 1.67b	42.76 ± 1.68a
	150 ppm	89.46	2	<0.05	17.58 ± 1.67c	29.78 ± 1.67b	49.82 ± 1.68a
	250 ppm	146.34	2	<0.05	18.33 ± 1.67c	35.00 ± 0.00b	64.65 ± 2.92a
	10 ppm	7.52	2	<0.05	6.67 ± 1.67b	11.67 ± 1.67ab	15.82 ± 1.68a
	20 ppm	13.88	2	<0.05	13.33 ± 1.67b	23.33 ± 1.67a	29.29 ± 2.92a
	30 ppm	28.95	2	<0.05	14.86 ± 1.67b	24.33 ± 1.67a	31.78 ± 1.68a
Z3	50 ppm	34.62	2	<0.05	18.33 ± 1.67c	26.67 ± 1.67b	34.34 ± 0.00a
	100 ppm	116.09	2	<0.05	18.33 ± 1.67c	26.67 ± 1.67b	52.86 ± 1.68a
	150 ppm	189.23	2	<0.05	20.67 ± 1.67c	28.33 ± 1.67b	59.32 ± 1.68a
	250 ppm	247.44	2	<0.05	23.33 ± 1.67c	33.33 ± 1.67b	73.06 ± 1.68a
	10 ppm	39.43	2	<0.05	11.67 ± 1.67c	21.67 ± 1.67b	32.66 ± 1.68a
	20 ppm	93.72	2	<0.05	18.33 ± 1.67c	28.33 ± 1.67b	44.44 ± 0.00a
	30 ppm	67.43	2	<0.05	22.88 ± 1.67c	32.67 ± 1.67b	49.86 ± 1.68a
Z4	50 ppm	89.45	2	<0.05	31.67 ± 1.67c	43.33 ± 1.67b	62.96 ± 1.68a
	100 ppm	42.37	2	<0.05	41.67 ± 1.67c	55.00 ± 2.89b	74.75 ± 2.92a
	150 ppm	89.75	2	<0.05	43.33 ± 1.67c	59.67 ± 1.67b	79.06 ± 1.68a
	250 ppm	109.47	2	<0.05	53.33 ± 1.67c	68.33 ± 1.67b	88.22 ± 1.68a
	10 ppm	53.49	2	<0.05	18.33 ± 1.67c	31.67 ± 1.67b	42.76 ± 1.68a
	20 ppm	39.00	2	<0.05	31.67 ± 1.67c	43.33 ± 1.67b	61.28 ± 1.68a
	30 ppm	63.06	2	<0.05	34.67 ± 1.67c	57.78 ± 1.67b	66.93 ± 1.68a
Z5	50 ppm	108.82	2	<0.05	43.33 ± 1.67c	58.33 ± 1.67b	78.11 ± 1.68a
	100 ppm	35.34	2	<0.05	68.33 ± 1.67c	78.33 ± 1.67b	88.22 ± 1.68a
	150 ppm	83.49	2	<0.05	72.86 ± 1.82c	82.96 ± 1.87b	93.43 ± 1.68a
	250 ppm	45.50	2	<0.05	81.67 ± 1.67c	91.67 ± 1.67b	100.00 ± 0.00a
	10 ppm	12.43	2	<0.05	2.34 ± 1.67b	3.02 ± 1.67a	3.89 ± 1.67a
	20 ppm	16.02	2	<0.05	2.89 ± 1.67b	3.98 ± 1.67a	4.25 ± 1.67a
*Azadirachta indica*	30 ppm	17.87	2	<0.05	3.56 ± 1.67b	4.08 ± 1.67a	4.78 ± 1.67a
	50 ppm	8.98	2	<0.05	6.06 ± 1.67b	8.21 ± 1.67a	9.32 ± 1.68a
	100 ppm	44.21	2	<0.05	8.23 ± 1.67b	8.95 ± 1.67b	11.63 ± 1.67a
	150 ppm	62.80.	2	<0.05	9.78 ± 1.67b	10.67 ± 1.67b	13.84 ± 1.68a
	250 ppm	78.27	2	<0.05	12.36 ± 1.67c	14.01 ± 1.67b	16.17 ± 1.68a
Permethrin	10 ppm	7.56	2	<0.05	2.32 ± 1.78c	5.67 ± 1.29b	13.51 ± 1.57a
	20 ppm	16.67	2	<0.05	3.85 ± 1.25c	8.92 ± 2.18b	18.01 ± 1.92a
	30 ppm	23.02	2	<0.05	9.43 ± 3.62c	22.46 ± 2.57b	39.28 ± 1.98a
	50 ppm	28.77	2	<0.05	12 ± 2.48c	29.84 ± 1.89b	43.36 ± 0.2.16a
	100 ppm	37.53	2	<0.05	19.27 ± 2.38c	35.17 ± 2.61b	51.95 ± 2.58a
	150 ppm	127.05	2	<0.05	23.63 ± 1.58c	41.21 ± 1.68b	67.14 ± 2.18a
	250 ppm	273.38	2	<0.05	29.32 ± 1.68c	52.89 ± 1.49b	76.46 ± 2.47a

^*^Convulvulus arvensis (Z1), *Chenopodium murale* (Z2), *Tribulus terrestris* (Z3), *Trianthema portulacastrum* (Z4), *Achyranthes aspera* (Z5), *Azadirachta indica* (Neem) and Permethrin.

### LC_50_ of weed plant extracts against *Culex quinquefasciatus* mosquitoes

*A. aspera* showed lowest LC_50_ (87, 39 and 9 ppm; p < 0.05) at 24, 48 and 72 hours, Similarly, low LC_50_ (205, 123.65 and 34.64; p < 0.05) was found with *T. portulacas* at 24, 48 and 72 hours, respectively; followed by *C. arvensis* (LC_50_ 304, 151, and 57; p < 0.05). *C. murale* showed highest LC_50_ (>250 ppm) at 24, 48 hours among all five extracts. High LC_50_ was observed with *T. terrestris* (>250 ppm) at 24, 48 hours, while it showed low LC_50_ (126 ppm; p < 0.05) but higher than LC_50_ (98 ppm; p < 0.05) of Permethrin at 72 hours. The LC_50_ of Permethrin was LC_50_ > 250 ppm and 247 ppm at 24 and 48 hours, respectively. Overall, it was found that all the weeds extracts showed significant results at 72 hours (p < 0.05) as compare to *A. indica* (Neem) extract, which showed LC_50_ (>>250 ppm) after all exposure time (p > 0.05; Table 3).

**Table 3:** Toxicity of weed plant extracts against *Culex quinquefasciatus*.

Treatment	Observation	N	LC50 (ppm) (Upper + lower values)	Slope ± SE	X2 ±	df	SE	P
Z1	24	100	LC50 > 250 ppm	0.0039830 ± 0.0006982	5.296	5	16.82	0.00
	48	100	151.0515 (125.8312 ± 185.6088)	0.0049704 ± 0.0006737	8.383	5	14.52	0.00
	72	100	57.1057 (37.0145 ± 75.4342)	0.0063345 ± 0.0007791	1.319	5	9.50	0.00
Z2	24	100	LC50 >> 250 ppm	0.0018983 ± 0.0007777	6.174	5	246.23	0.32
	48	100	LC50 > 250 ppm	0.0018983 ± 0.0007777	6.174	5	53.40	0.10
	72	100	164.9892 (140.0672 ± 199.3816)	0.0053894 ± 0.0006789	3.451	5	14.47	0.05
Z3	24	100	LC50 >> 250 ppm	0.0020102 ± 0.0007382	4.355	5	184.87	0.22
	48	100	LC50 >> 250 ppm	0.0019029 ± 0.0006802	6.097	5	133.47	0.10
	72	100	126.5443 (106.1374 ± 151.5917)	0.0058605 ± 0.0006952	8.582	5	11.22	0.03
Z4	24	100	205.0356 (169.5248 ± 262.854)	0.0043157 ± 0.0006678	12.09	5	22.03	0.03
	48	100	123.6593 (99.0462 154.7968)	0.0046799 ± 0.000674	9.695	5	13.55	0.00
	72	100	34.6413 (13.5864 ± 51.9514)	0.0069505 ± 0.0008466	11.74	5	9.45	0.00
Z5	24	100	87.4652 (70.6095 ± 105.3030)	0.0069648 ± 0.0007523	17.60	5	8.64	0.01
	48	100	39.0875 (21.3783 ± 54.3348)	0.0078991 ± 0.0009073	10.63	5	8.1687	0.00
	72	100	9.2235 (6.3036 ± 19.7265)	0.014552 ± 0.002114	5.608	5	6.2459	0.00
	24	100	616.028(429.372 ± 1279.82)	0.0029602 ± 0.0008561	2.577	5	147.95	0.75
*A. indica* Neem	48	100	600.278(420.494 ± 1220.25)	0.0028745 ± 0.0008160	2.54713	5	141.535	0.76
	72	100	546.163(394.025 ± 1005.21)	0.0029899 ± 0.0007771	3.86339	5	116.025	0.56
Permethrin	24	100	LC50 > 250 ppm	0.03432162 ± 0.0065762	14.2512	6	15.7262	0.03
	48	100	247.0248 (179.6136 ± 298.7321)	0.0042325 ± 0.00071697	8.1245	6	13.23	0.02
	72	100	98.2859 (86.7189 ± 145.4137)	0.0068465 ± 0.00082418	5.82	6	7.49	0.01

^*^Convulvulus arvensis (Z1), *Chenopodium murale* (Z2), *Tribulus terrestris* (Z3), *Trianthema portulacastrum* (Z4), *Achyranthes aspera* (Z5), *Azadirachta indica* (Neem) and Permethrin.

### Mortality Assay of *Culex quinquefasciatus* mosquitoes using *Achyrathes aspera* in-combination with microbes at various concentration and different time intervals

*A. aspera* showed significant results for mortality and thus, it was selected for combinatorial trials with *Bti* and *P. aeruginosa*. The mean mortality induced by *Bti* and *Pseudomonas* individually and in combinations with *A. aspera* extract at different concentrations and time intervals is shown in Table 4. It was observed that mortality increased with increase in concentration and exposure time. High mortality was observed at 250 ppm concentration after 72 hours, whereas, highest mortality (100%) was shown by of *A. aspera* with *Bti* followed by combination of *Pseudomonas* and *A. aspera* (72.79%) at 250ppm concentration as compared to control treatment (Table 4).

**Table 4:** Mean mortality of *Culex quinquefasciatus* using *Achyranthes aspera* with microbes at various concentration and different time intervals.

Treatment	Conc.	F Value	df	P value	Mean mortality with different time intervals		
					24 hours	48 hours	72 hours
	10 ppm	27.81	2	<0.05	12.46 ± 1.68c	20.07 ± 1.70b	30.27 ± 1.70a
	20 ppm	16.90	2	<0.05	22.56 ± 1.68c	33.67 ± 2.95b	43.88 ± 2.95a
	30 ppm	30.67	2	<0.05	24.86 ± 1.68c	35.48 ± 1.70b	45.63 ± 1.70a
*Bti*	50 ppm	39.90	2	<0.05	30.98 ± 1.68c	42.18 ± 1.70b	52.38 ± 1.70a
	100 ppm	35.34	2	<0.05	42.76 ± 1.68c	50.68 ± 1.70b	59.18 ± 0.00a
	150 ppm	31.61	2	<0.05	45.24 ± 1.68c	53.67 ± 1.70b	63.58 ± 1.70a
	250 ppm	34.51	2	<0.05	51.18 ± 1.68c	60.88 ± 1.70b	71.09 ± 1.70a
*Bti* + Z5	10 ppm	77.64	2	<0.05	17.51 ± 1.68c	30.27 ± 1.70b	47.28 ± 1.70a
	20 ppm	46.87	2	<0.05	30.98 ± 1.68c	43.88 ± 2.95b	60.88 ± 1.70a
	30 ppm	43.58	2	<0.05	35.28 ± 1.68c	48.33 ± 2.57b	65.45 ± 1.70a
	50 ppm	39.33	2	<0.05	42.76 ± 1.68c	55.78 ± 3.40b	72.79 ± 1.70a
	100 ppm	54.70	2	<0.05	54.55 ± 2.92c	69.39 ± 2.95b	89.80 ± 0.00a
	150 ppm	98.19	2	<0.05	57.72 ± 1.68c	74.68 ± 1.70b	92.53 ± 1.70a
	250 ppm	183.90	2	<0.05	62.96 ± 1.68c	86.39 ± 1.70b	100.00 ± 0.00a
*Pseudomonas*	10 ppm	7.25	2	<0.05	1.35 ± 1.35b	4.76 ± 1.70ab	9.86 ± 1.70a
	20 ppm	41.06	2	<0.05	4.04 ± 0.00c	11.56 ± 1.70b	21.77 ± 1.70a
	30 ppm	78.54	2	<0.05	4.89 ± 0.00c	11.93 ± 1.70b	23.91 ± 1.70a
	50 ppm	52.23	2	<0.05	7.41 ± 1.68c	13.27 ± 0.00b	26.87 ± 1.70a
	100 ppm	41.51	2	<0.05	14.14 ± 0.00c	21.77 ± 1.70b	31.97 ± 1.70a
	150 ppm	63.46	2	<0.05	17.26 ± 1.68c	23.45 ± 1.70b	36.62 ± 1.70a
	250 ppm	83.41	2	<0.05	22.56 ± 1.68c	30.27 ± 1.70b	52.38 ± 1.70a
*Pseudomonas* + *Bti*	10 ppm	15.50	2	<0.05	4.04 ± 0.00c	9.86 ± 1.70b	14.97 ± 1.70a
	20 ppm	55.10	2	<0.05	7.41 ± 1.68c	14.97 ± 1.70b	31.97 ± 1.70a
	30 ppm	86.98	2	<0.05	9.04 ± 1.68c	16.63 ± 1.70b	33.47 ± 1.70a
	50 ppm	46.23	2	<0.05	12.46 ± 1.68c	21.77 ± 1.70b	35.37 ± 1.70a
	100 ppm	33.51	2	<0.05	22.56 ± 1.68c	31.97 ± 1.70b	42.18 ± 1.70a
	150 ppm	98.34	2	<0.05	26.77 ± 1.68c	36.52 ± 1.70b	49.32 ± 1.70a
	250 ppm	150.66	2	<0.05	32.66 ± 1.68c	43.88 ± 0.00b	65.99 ± 1.70a
*Pseudomonas* + Z5	10 ppm	39.25	2	<0.05	2.69 ± 1.35c	9.86 ± 1.70b	18.37 ± 0.00a
	20 ppm	46.04	2	<0.05	7.41 ± 1.68c	16.67 ± 1.70b	30.27 ± 1.70a
	30 ppm	28.92	2	<0.05	10.63 ± 1.68c	21.34 ± 1.70b	36.82 ± 1.70a
	50 ppm	36.17	2	<0.05	15.82 ± 1.68c	28.57 ± 2.95b	42.18 ± 1.70a
	100 ppm	52.50	2	<0.05	27.61 ± 1.68c	40.48 ± 1.70b	59.18 ± 2.95a
	150 ppm	47.48	2	<0.05	31.23 ± 1.68c	46.59 ± 1.70b	64.34 ± 2.95a
	250 ppm	58.84	2	<0.05	36.03 ± 1.68c	52.38 ± 1.70b	72.79 ± 3.40a

^*^Achyranthes aspera (Z5), *Psuedomonas*, *Bacillus thuringiens isisraeliensis* (*Bti*).

### LC_50_ of *Achyrathes aspera* extract in-combination with microbes against *Culex quinquefasciatus* mosquitoes

The combination of *A. aspera* and *Bti* showed low LC_50_ (137, 49 and 8 ppm; p < 0.05) at 24, 48 and 72 h, respectively. However, the combination of *Pseudomonas* and *A. aspera* showed very high LC_50_ (>250 ppm) at 24 h and, moderate to high LC_50_ (213 and 111 ppm; p > 0.05) was found at 48 and 72 h, respectively. For control treatment, the LC_50_ of *Bti* (213.45, 145.80 and 76.12 ppm; p < 0.05) was found lower than *Pseudomonas* (>250 ppm) at 24, 48 and 72 h, respectively. The LC_50_ of combination of *Pseudomonas* and *Bti* is shown in Table 5.

**Table 5:** Toxicity of *Achyranthes aspera* extract in combination with microbes against *Culex quinquefasciatus*.

Treatment	Observation	N	LC50 (ppm) (Upper ± lower values)	Slope ± SE	X2 ±	df	SE	P value
*Bti*	24	100	213.4507 (172.9759 ± 284.6938)	0.0038483 ± 0.0006629	11.840	3	25.789	0.00
	48	100	145.8052 (113.6218 ± 194.4482)	0.0036579 ± 0.0006575	10.203	3	18.893	0.01
	72	100	76.1231 (42.6566 ± 107.7533)	0.0036931 ± 0.0006723	7.694	3	15.507	0.05
*Bti* ± Z5	24	100	137.0498 (109.2724 ± 175.2752)	0.0042117 ± 0.0006644	16.361	3	15.810	0.00
	48	100	49.6853 (27.9094 ± 68.5992)	0.0061320 ± 0.0007723	8.902	3	10.034	0.00
	72	100	8.2924 (−7.7614 ± 18.9967)	0.014400 ± 0.002129	1.475	3	6.4015	0.00
*Pseudomonas*	24	100	LC50 > 250 ppm	0.0044863 ± 0.0008278	5.935	3	53.815	0.11
	48	100	LC50 > 250 ppm	0.0034591 ± 0.0007267	5.432	3	61.388	0.14
	72	100	226.9449 (187.1139 ± 294.4419)	0.0042459 ± 0.0006707	6.336	3	25.155	0.09
*Pseudomonas* + *Bti*	24	100	LC50 > 250 ppm	0.0044517 ± 0.0007379	7.302	3	39.260	0.06
	48	100	LC50 > 250 ppm	0.0039967 ± 0.0006806	5.565	3	32.651	0.13
	72	100	153.6023 (127.0986 ± 190.8331)	0.0047483 ± 0.0006711	7.794	3	15.399	0.05
*Pseudomonas* + Z5	24	100	LC50 > 250 ppm	0.0047247 ± 0.0007258	16.513	3	32.242	0.00
	48	100	213.0870 (177.4763 ± 270.5488)	0.0044985 ± 0.0006722	11.693	3	22.067	0.03
	72	100	111.5831 (90.7470 ± 135.9624)	0.0055370 ± 0.0006938	13.540	3	11.147	0.68

^*^Achyranthes aspera (Z5), *Psuedomonas*, *Bacillus thuringiens isisraeliensis* (*Bti*).

### Enzyme inhibitory effect of weed plant extracts in *Culex quinquefasciatus* mosquitoes

Maximum inhibition activity of AChE, AcP, AkP, α-Carboxyl and β-Carboxyl was found for *A. aspera* as 54.68, 34.28, 24.58, 71.08 and 59.96 at 250 ppm, respectively. The mean value for inhibition activity for aforementioned mentioned enzymes by *C. arvensis* was found 52.94, 32.02, 21.01, 63.88 and 46.91%, respectively; followed by *C. murale* as 59.95, 29.98, 26.08, 45.04 and 50.01% at 250 ppm concentration. Similalry, *T. terrestris* showed inhibition of AChE, AcP, AkP, α-Carboxyl and β-Carboxyl as 48.78, 28.16, 25.02, 47.13 and 47.04, respectively; likewise, *T. portulacastrum* showed as 51.14, 29.08, 24.98, 47.68 and 49.69, respectively at 250 ppm. Overall, the enzyme assay showed that the inhibition of enzyme activity increased with increase of concentration (Table 6).

**Table 6:** Percent inhibition of enzyme activity in *Culex quinquefasciatus* larvae using different concentrations of *Bti* at 30% concentrations.

Plants	Concentration	AChE	AcP	AkP	α-Carboxyl	β-Carboxyl
**Z1**	50 ppm	14.85 ± 2.83 a	14.90 ± 1.75 a	8.35 ± 1.08 a	30.01 ± 2.02 a	26.65 ± 2.23 a
	100 ppm	31.14 ± 3.36 b	21.07 ± 1.43 b	12.95 ± 0.78 a	52.80 ± 3.01 b	43.87 ± 3.34 b
	250 ppm	52.94 ± 2.43 c	32.02 ± 1.14 c	21.01 ± 1.57 b	63.88 ± 1.67 c	46.91 ± 3.08 b
	F, df and P value	(F = 92.99; df = 2; P < 0.05)	(F = 32.03; df = 2; P < 0.05)	(F = 24.56 df = 2; P < 0.05)	(F = 78.92; df = 2; P < 0.05)	(F = 23.02; df = 2; P < 0.05)
**Z2**	50 ppm	17.83 ± 2.3 a	15.77 ± 1.43 a	8.87 ± 1.16 a	24.08 ± 2.13 a	26.13 ± 3.38 a
	100 ppm	31.12 ± 3.32 b	20.97 ± 1.03 b	12.91 ± 0.93 b	33.10 ± 4.11 a	39.78 ± 3.03 b
	250 ppm	59.95 ± 2.47 c	29.98 ± 1.23 c	26.08 ± 0.69 c	45.04 ± 3.513b	51.01 ± 2.57 b
	F, d.f and P value	(F = 90.86; df = 2; P < 0.05)	(F = 38.79; df = 2; P < 0.05)	(F = 128.87; df = 2; P < 0.05)	(F = 13.05; df = 2; P < 0.05)	(F = 14.03; df = 2; P < 0.05)
**Z3**	50 ppm	11.93 ± 2.69 a	19.61 ± 1.38 a	9.49 ± 1.19 a	22.93 ± 2.82 a	16.18 ± 3.74 a
	100 ppm	24.03 ± 2.59 b	21.83 ± 1.68 a	16.09 ± 1.39 b	36.15 ± 3.08 b	36.24 ± 3.49 b
	250 ppm	48.78 ± 1.82 c	28.16 ± 1.24 b	25.02 ± 1.29 c	47.13 ± 3.39 c	47.04 ± 2.52 b
	F, d.f and P value	(F = 59.57; df = 2; P < 0.05)	(F = 12.89; df = 2; P < 0.05)	(F = 39.94; df = 2; P < 0.05)	(F = 23.29; df = 2; P < 0.05)	(F = 23.31; df = 2; P < 0.05)
**Z4**	50 ppm	20.67 ± 2.87 a	19.96 ± 1.49 a	11.03 ± 1.49 a	28.49 ± 2.59 a	22.03 ± 4.87 a
	100 ppm	27.79 ± 2.39 a	23.21 ± 1.54 b	13.48 ± 1.58 a	45.08 ± 2.78 b	35.03 ± 3.61 ab
	250 ppm	51.14 ± 2.78 b	29.08 ± 1.29 c	24.98 ± 1.38 b	47.68 ± 3.71 b	49.69 ± 3.89 b
	F, d.f and P value	(F = 45.89; df = 2; P < 0.05)	(F = 42.39; df = 2; P < 0.05)	(F = 33.54; df = 2; P < 0.05)	(F = 12.89; df = 2; P < 0.05)	(F = 11.23; df = 2; P < 0.05)
**Z5**	50 ppm	20.69 ± 2.03 a	18.07 ± 1.08 a	10.17 ± 0.74 a	37.93 ± 2.11 a	31.01 ± 2.93 a
	100 ppm	38.76 ± 3.06 b	23.29 ± 1.39 b	15.34 ± 0.74 b	58.17 ± 2.89 b	48.16 ± 3.04 b
	250 ppm	54.68 ± 1.35 c	34.28 ± 1.13 c	24.58 ± 1.14 c	71.08 ± 3.03 c	59.96 ± 1.68 c
	F, d.f and P value	(F = 59.24; df = 2; P < 0.05)	(F = 47.87; df = 2; P < 0.05)	(F = 51.02; df = 2; P < 0.05)	(F = 52.23; df = 2; P < 0.05)	(F = 31.07; df = 2; P < 0.05)

*Convulvulus arvensis* (Z1), *Chenopodium murale* (Z2), *Tribulus terrestris* (Z3), *Trianthema portulacastrum* (Z4) and *Achyranthes aspera* (Z5).

AChE = acetylcholinesterase, AcP = acid phosphatases, AkP = alkaline phosphatases, α-Carboxyl = α-Carboxylesterases and β-Carboxyl = β-Carboxylesterases.

Means sharing the same letter within each treatment is not statistically different.

### Phytochemical constituents in weed extracts

Phytochemical analysis revealed the presence of flavonoids, saponins, tannins, steroids, cardiac glycosides, alkaloids, anthrequinones and terpenoids in all the five weeds extracts used against *Cx. quiquefasciatus* larvae (Table 7).

**Table 7:** The chemical constituents present in five weeds extract.

Sr. No.	Code#	Weed Plants	Chemical Constituents							
			Fl	Sa	Tn	St	CG	Al	Anth	Ter
1	Z1	*C. arvensis*	+	+	+	−	+	+	+	+
2	Z2	*C.muale*	+	+	+	+	+	+	−	−
3	Z3	*T. terrestris*	+	+	+	+	−	+	+	+
4	Z4	*T. portulacastrum*	+	+	+	+	+	+	+	+
5	Z5	*A.aspera*	+	+	+	+	+	+	+	+

^*^Convulvulus arvensis (Z1), *Chenopodium murale* (Z2), *Tribulus terrestris* (Z3), *Trianthema portulacastrum* (Z4) and *Achyranthes aspera* (Z5).

^*^Fl = flavonoids, Sa = saponins, Tn= tannins, St = steroids, CG = Cardiac glycosides, Al= alkaloids, Anth= anthrequinones and Ter = terpenoids.

### Fourier transform infrared spectroscopy (FTIR) analysis of *Achyranthes aspera* extract

**Figure 1:**
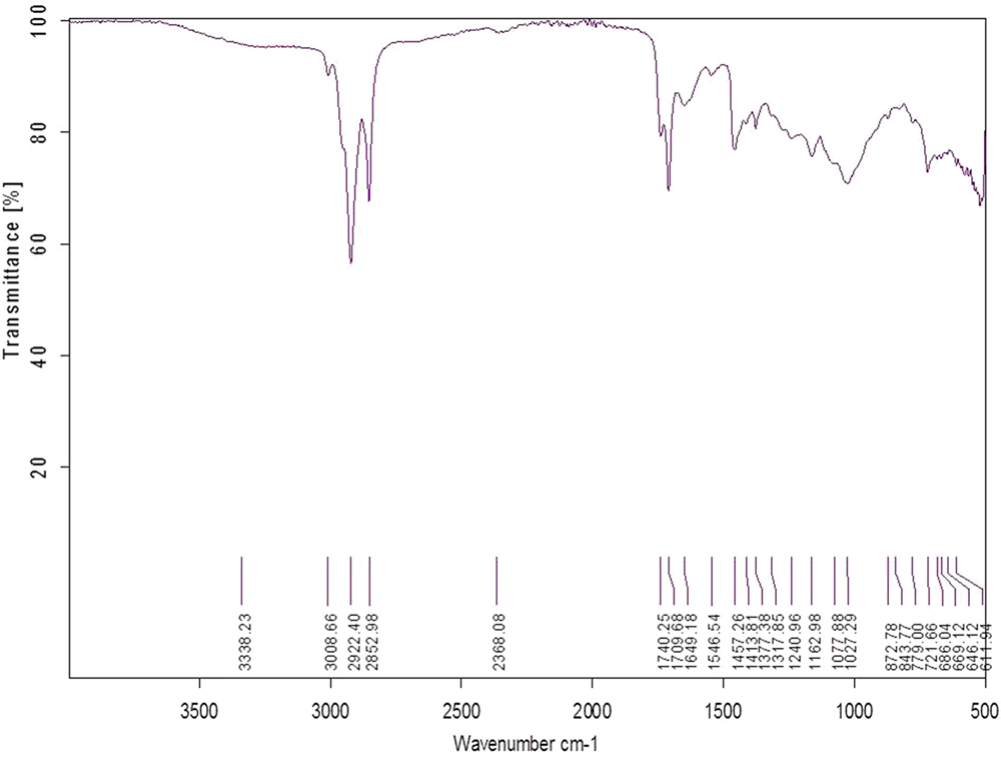
Full FTIR spectrum of *Achyranthes aspera*. The spectrum shows a range of 4000 to 400 cm^−1^ wave number (along X-axis) the function of percent (%) transmittance (along Y-axis). The peaks observed at 669.12 cm^−1^ correspond to aromatic ring having phosphate group, 779.00 cm^−1^ out of plane CH bending, 1077.88 cm^−1^ C-O Stretching, 1317.85 cm^−1^ and 1377.38 cm^−1^ C=H group, 3008.66 cm^−1^ alkyl C-H group stretching and 3338.23 cm^−1^–OH group for phenols.

The Infra-red spectrum of *A. aspera* extract revealed peak at 3338. 23 cm^−1^ which corresponds to –OH group for phenols. Moreover, peak at around 1317.85 cm^−1^ and 1377.38 cm^−1^ was assigned to C=H group and 1077.88 cm^−1^ for C-O Stretching. The peak at 779.00 cm^−1^ shows the stretching vibration of plane C-H bending. In addition, the peak at 669.12 cm^−1^ corresponds to aromatic ring having phosphate group (Fig. 1). Hence, the FTIR results showed the presence of phenolic compound in extract of *A. aspera*.

### Random amplified polymerase DNA polymerase chain reaction (RAPD-PCR) analysis

**Figure 2:**
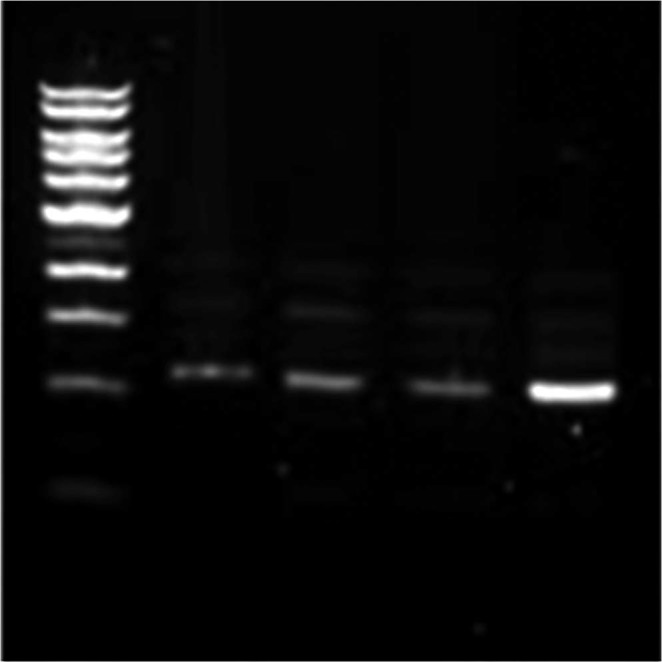
Comparison of RAPD profile for genotoxicity using C-04 primer. (1) Control group (Non-treated *Cx. quiquefasciatus* larvae). (2) Treated larvae with *A. aspera* extract. (3) Treated larvae with *Bti*. (4) The combination of *A. aspera* with *Bti*.

The extracted DNA from larvae of *Cx. quiquefasciatus* (treated with *Achyrathes aspera* and its combination with *Bti*) was amplified using RAPD-PCR. The banding profile showed that no DNA damage had occurred due to application of *A. aspera* individually and in combination with *Bti* compared to control (Fig. 2).

### Comet assay

**Figure 3:**
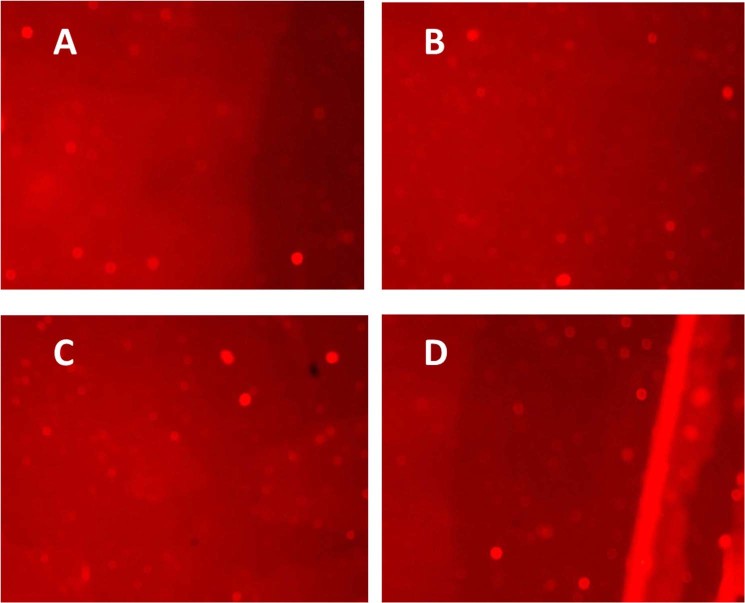
Comparison of Comet assay profile for genotoxicity in larvae of *Culex quiquefasciatus*. (**A**) Control. (**B**) *A. aspera*. (**C**) *Bti*. (**D**) *A. aspera* + *Bti*.

The comet assay was performed to detect the DNA damage in cells of treated larvae of *Cx. quiquefasciatus* with significant plant extract *A. aspera* and its combination with *Bti*. It was found that no DNA damage had occurred in treatment group compared to control (Fig. 3).

## Discussions

During the present study, weed plant extracts were exploited for their insecticidal potential in *Cx. quiquefasciatus*. Five different weed plants extracts *C. arvensis*, *C. murale*, *T. terrestris*, *T. portulacastrum* and *A. aspera* were employed against larvae of *Cx. quiquefasciatus*. The extracts of three weed plants viz. *A. aspera, T. portulacastrum* and *C. arvensis* showed significant results as compare to synthetic pesticide Permethrin, and in comparison with *A. indica* (neem) extract all plants showed high mortality. *A. indica* extract had already been reported by Sagheer *et al*.^14^ which showed 16.11% mortality at 15% concentration. Recently, Sultana^49^ and Sultana *et al*.^15^ described that weed plant extracts have more insecticidal activity as compared to *A. indica* extracts. Subsequently, *A. aspera* revealed LC_50_ value 9.22 ppm with 100% mortality which is lower than that of *Clausena dentate* (LC_50_ 28.60 ppm) against *Cx. quinquefasciatus* larvae as described by Sakthivadivel *et al*.^50^. Similarly, it was also revealed that *A. aspera* had more entomocidal potential when compared the LC_50_ 89.03 ppm of *Croton rhamnifolioides* as reported by Santos *et al*.^51^.

In current study, all the plant extracts showed high mortality at 72 hours compared to 24 and 48 hours. And no LC_50_ was found more than 250 ppm (LC_50_ < 250 ppm) at 72 hours exposure time. Thus, the insecticidal activity of weed plant extracts is time dependent^15^. It was thus found that the mortality had increased with increased concentrations of weed plant extracts. Hence, the percent mortality and toxicity data is in accordance to the previous findings of Odeyemi and Ashamo^52^, Sultana *et al*.^15^ and Sagheer *et al*.^14^ that the plant extracts become more toxic with increased dose and exposure time. Interestingly, few LC_50_ values in the current findings were extrapolated which are in line with study of Prabakar and Jebanesan^53^ who reported LC_50_ of *Benincasa cerifera* (LC_50_ = 1189.30 ppm) and *Citrullus vulgaris* (LC50 = 1636.04 ppm) and found higher LC_50_ than 1000 ppm (maximum concentration)^53^.

Plant extracts have also been used in combination with certain microbes. Kumar *et al*.^54^ described that *B. thuringiensis* in combination with *Solanum xanthocarpum* showed higher larval mortality^54^. Recently, *B. thuringiensis* was reported as very effective causing high mortality when used in combination with weed plant extracts^49^. In addition, Kupferschmied *et al*.^28^ extensively reviewed the root-associated *Pseudomonas* with their insecticidal activities against various insect pests. Consistently, the current findings showed that *A. aspera* in combination with *B. thuringiensis* had caused 100% mortality against larvae of *Cx. quinquefasciatus*.

Phytochemical analysis of the weed plant extracts used in present study indicated the presence of flavonoids. It has been reported by Gautam *et al*.^55^ that flavonoid extracted from aerial parts of *Androgrpahis paniculata* was inactive at 600 ppm against *Ae. aegypti* larvae, but it caused 70% mortality against *An. stephensi* at 200 ppm concentration. However, flavonoid extracted from flower-buds showed 100% mortality for *Ae. aegypti* and *An. stephensi* at 600 & 200 ppm concentration, respectively. *A. aspera* weed plant contains flavonoids, saponins, tannins, steroids, cardiac glycosides, alkaloids, anthrequinones and terpenoids. These compounds are also present in other tested plants, except steroids and cardiac glycosides which were found absent in *C. arvensis* and *T. terrestris*. Similarly, anthrequinones and terpenoids were also found absent in *C. muale*. Low mortality was shown by *C. muale* in the current study which might be due to the absence of anthrequinones and terpenoids, and absence of steroids in case of *T. terrestris*.

Subsequent studies by Cárdenas-Ortega *et al*.^20^ reported 37 different compounds with β-caryophyllene and caryophyllene oxide as main components in *Salvia ballotiflora* resulted in 80% larval mortality. Evans^56^ reported that alkaloids cause the death of treated organisms due to their ability to bind DNA of organisms and affecting the replication process and synthesis of molecules. Alkaloids compound were identified in all of our used weed plants extracts but no DNA damage was observed in the treated larvae of *Cx. quinquefasciatus* mosquiotes. The enzymatic profiles are also modulated in response to natural oils from plants^25^. For instance, Esterases, a major detoxifying enzyme in insects and have been reported to involve in detoxification of insecticides^26^. Plant extracts have been reported as AChE inhibitors^27^. The death in insects due to treatment with plant extracts suggested that the molecules present therein possibly interfere at the cholinergic synapse and destroyed the communication network from one exonic end to another; thereby, blocking the nerve impulse transmission. Thus, the lethal effect may also be due to the accumulation of acetylcholine (ACh), a neurotransmitter, at synaptic junctions, which interrupts the coordination between the nervous and muscular junctions (neurotoxicity)^27^. Subsequent changes in enzyme activity are also reported for Phosphatases in insects. The hydrolysis of acid phosphatase (ACP) and alkaline phosphatase (ALP) phosphomonoesters under acid or alkaline conditions, respectively^25^. Alkaline phosphatase (ALP) is used as a membrane marker enzyme, active in intestinal epithelial cells, malpighian tubules and hemolymph of insects^25,57^. A decrease in ACP levels due to plant extract could be attributed to reduced phosphorous liberation for energy metabolism, decreased rate of metabolism as well as decreased rate of transport of metabolites^25,26^. The enzyme activity of AChE, AcP, AkP, α-Carboxyl and β-Carboxyl was inhibited with increase of concentration of all the plant extracts which is also in agreement to Santos *et al*.^51^ who also reported that essential oil of *Croton rhamnifolioides* showed the inhibitory effect on a digestive enzyme (Trypsin) from larvae of *Ae. aegypti*.

The genotoxicity and carcinogenicity in the cell genome are caused by genotoxic agents having some lethal or sub-lethal effects which are induced by some xenobiotic substances^22,26^.

Thus, the DNA damage due to exposure of an organism to plant extracts^21,22^ may result from the formation of covalently bound adducts between metabolites and DNA; and the faulty repair of these adducts often results in mutations and sometimes cytogenetic changes. Recently *A. aspera* was found genotoxic against *Ae. aegypti* with significant changes in the RAPD profiles. These changes suggested that certain phytocomponents in *A. aspera* caused the probable DNA damage and mutations in the larval g-DNA which could be the possible reason of larval mortality^24^. In contrast, no DNA damage was found in the current findings. Moreover, FTIR analysis of *A. aspera* indicated the presence of phytochemicals composed of hydrogen bonded –OH functional group. Mostly phenolic phytochemicals such as tannins and flavonoids are composed of –OH functional group^58^. FTIR spectrum of *C. arvensis* showed the presence of alkaloids. These all compounds are reported as toxic to insects and produced insecticidal activities. It has already been reported that phenolic compounds can be potentially used for the control of insect pests of various crops^59^.

Hence, it is suggested that the mortality in larvae of *Cx. quinquefasciatus* cannot be attributed due to genotoxicity. Rather, perhaps it is caused by the presence of certain phenolic phytochemicals such as flavonoids which modulate the enzymatic activity and thus, cause the death of larvae of *Cx. quinquefasciatus*. Thus, it is suggested that *A. aspera* weed plant can be further exploited for extraction and purification of phenolic compounds to use against mosquitoes. In addition, the current study which is the first one performed in Pakistan using weed plants extracts; also suggests that weed plants can be explored for their insecticidal activity against other insect pests.

## Conclusions

The petroleum ether extracts of five weed plants were used against the larvae of *Cx. quinquefasciatus. A. aspera* extract showed highest mortality. Thus, based on LC_50_ values (p-values), *Achyrathes aspera* weed plant extract was used along with *Bti* and *Pseudomonas* bacteria for further trials. The highest mortality of *C. quinquefasciatus* was found using *A. aspera* with *Bti*. Enzyme inhibition activity of AChE, AcP, AkP, α-Carboxyl and β-Carboxyl was found in tested weed plant extracts. Phytochemical analysis showed the presence of flavonoids, saponins, tannins, steroids, cardiac glycosides, alkaloids, anthrequinones and terpenoids. Moreover, FTIR analysis showed that *A. aspera* contains phenolic compounds which have been reported to show insecticidal activity. Genotoxic activity was also observed using RAPD-PCR and comet assay. It was found that no DNA damage had been occurred due to either *A. aspera* extractor using the extract in combination with *Bti*. It is suggested that certain phenolic compounds such as flavonoids which modulate the enzymatic activity and, causes the death of larvae of *Cx. quinquefasciatus*. *A. aspera* plant is easily available in Pakistan and its extract could be very used to control *Culex* mosquitoes. In future, further studies are needed to extract and characterize the particular potential of phenolic compound found in *A. aspera* to use in mosquito control programs.

## Supplementary information

Supplementary Dataset .
