# Supplementary material for: Insecticidal and Genotoxic effects of some indigenous plant extracts in Culex quinquefasciatus Say Mosquitoes
Source: Sci Rep. 2020 Apr 22;10:6826. doi: 10.1038/s41598-020-63815-w (PMC7176662; doi:10.1038/s41598-020-63815-w)
Supplement: Supplementary file 1 — Supplementary Dataset . [file 41598_2020_63815_MOESM1_ESM.docx]

**Insecticidal and Genotoxic effects of some indigenous plant extracts in *Culex quinquefasciatus* Say Mosquitoes**

Muhammad Zulhussnain, Muhammad Kashif Zahoor*, Hina Rizvi^1^, Muhammad Asif Zahoor^2^, Azhar Rasul, Aftab Ahmad^3^, Humara Naz Majeed^4^, Amer Rasul^5^, Kanwal Ranian and Farhat Jabeen

Department of Zoology, Government College University Faisalabad, Pakistan

^1^Department of Environmental Sciences & Engineering, Government College University Faisalabad, Pakistan

^2^Department of Microbiology, Government College University Faisalabad, Pakistan

^3^Centre of Department of Biochemistry/US-Pakistan Center for Advance Studies in Agriculture and Food Security (USPCAS-AFS), University of Agriculture Faisalabad

^4^Department of Biochemistry, Government College Women University, Faisalabad, Pakistan

^5^Department of Entomology, University of Agriculture Faisalabad

###### **Dr. Muhammad Kashif Zahoor (Corresponding Author)**

Entomology Lab, Department of Zoology, Government College University, Faisalabad.

Phone: +92 41 9201206; Mob: 0092-333-7664700, [kashif.zahoor@gcuf.edu.pk](mailto:kashif.zahoor@gcuf.edu.pk)

**Supplementary Table:**. Primers used for RAPD-PCR analysis

| **Sr. No.** | **Primers** | **Nucleotide sequence** |
| --- | --- | --- |
| 1 | A-03 | 5'- AGTCAGCCAC -3' |
| 2 | A-04 | 5'- AATCGGGCTG -3' |
| 3 | A-06 | 5'- GGTCCCTGAC -3' |
| 4 | A-18 | 5'- AGGTGACCGT -3' |
| 5 | C-04 | 5'- CCGCATCTAC -3' |
